# Development and Testing of a Method for Validating Chemical Inactivation of Ebola Virus

**DOI:** 10.3390/v10030126

**Published:** 2018-03-13

**Authors:** Kendra J. Alfson, Anthony Griffiths

**Affiliations:** Department of Virology and Immunology, Texas Biomedical Research Institute, San Antonio, TX 78227, USA; kalfson@txbiomed.org

**Keywords:** Ebola virus, chemical inactivation, viability testing, limit of detection, cytopathic effect

## Abstract

Complete inactivation of infectious Ebola virus (EBOV) is required before a sample may be removed from a Biosafety Level 4 laboratory. The United States Federal Select Agent Program regulations require that procedures used to demonstrate chemical inactivation must be validated in-house to confirm complete inactivation. The objective of this study was to develop a method for validating chemical inactivation of EBOV and then demonstrate the effectiveness of several commonly-used inactivation methods. Samples containing infectious EBOV (*Zaire ebolavirus*) in different matrices were treated, and the sample was diluted to limit the cytopathic effect of the inactivant. The presence of infectious virus was determined by assessing the cytopathic effect in Vero E6 cells. Crucially, this method did not result in a loss of infectivity in control samples, and we were able to detect less than five infectious units of EBOV (*Zaire ebolavirus*). We found that TRIzol LS reagent and RNA-Bee inactivated EBOV in serum; TRIzol LS reagent inactivated EBOV in clarified cell culture media; TRIzol reagent inactivated EBOV in tissue and infected Vero E6 cells; 10% neutral buffered formalin inactivated EBOV in tissue; and osmium tetroxide vapors inactivated EBOV on transmission electron microscopy grids. The methods described herein are easily performed and can be adapted to validate inactivation of viruses in various matrices and by various chemical methods.

## 1. Introduction

An increasing number of zoonotic pathogens, particularly RNA viruses, are important targets for research because of their potential to emerge in new hosts or regions [1,2]. However, such research can be challenging when working with viruses that must be handled in maximum containment laboratories. For example, Ebola virus (EBOV) is a highly lethal RNA virus that can cause Ebola virus disease (EVD) [3]. No approved vaccines or therapies exist for EBOV infections and the virus must be handled in Biosafety Level 4 (BSL4) laboratories. Within the United States (U.S.), these laboratories are regulated by the U.S. Centers for Disease Control and Prevention’s (CDC) Division of Select Agents and Toxins (DSAT) and the U.S. Department of Agriculture’s (USDA) Animal and Plant Health Inspection Service (APHIS).

Complete inactivation of infectious EBOV, or other regulated pathogens, is required before a sample may be removed from a BSL4 laboratory for analysis at a lower containment level. To comply with U.S. Federal Select Agent Program (FSAP) regulations (7 CFR Part 331, 9 CFR Part 121.3, 42 CFR Part 73.3), any select agent must be rendered non-infectious prior to removal from Select Agent registered space. For infectious agents, this requires that the agent no longer have any ability to grow or replicate. This can be achieved by a chemical inactivation procedure, many of which are well established, but the procedure must be appropriately validated in-house to confirm a lack of infectivity [4]. However, many of the chemicals used for routine inactivation are cytotoxic [5], which makes confirming inactivation challenging when the readout requires a healthy cell. Thus, there is a need to use an in vitro method that allows for demonstration of a lack of infectious virus in the entirety of a potentially inactivated sample without causing excessive cytotoxicity.

The objectives of this study were to develop a method for validating chemical inactivation procedures and to apply the method to demonstrate the effectiveness of several commonly-used inactivation methods. Material from samples routinely generated inside the BSL4 laboratory were used to validate inactivation of EBOV using various chemical methods, including TRIzol reagent (Life Technologies, Carlsbad, CA, USA), TRIzol LS reagent (Life Technologies), 10% neutral buffered formalin (Sigma-Aldrich, Saint Louis, MO, USA), RNA-Bee reagent (Tel-test, Friendswood, TX, USA) and osmium tetroxide (Sigma-Aldrich). To demonstrate a lack of infectious virus after inactivation, potentially inactivated material was passaged through two blind serial passages on Vero E6 cells, and the presence of infectious virus was determined by assessing cytopathic effects (CPE) and green fluorescence when possible. To demonstrate the sensitivity of this method, a control assay was performed that detected less than five plaque-forming units (PFU). All of the methods tested were shown to be effective at inactivating EBOV (*Zaire ebolavirus*, Kikwit variant).

## 2. Materials and Methods

### 2.1. Ethics Statement

To validate inactivation of samples from nonhuman primates (NHPs) exposed to EBOV, serum and tissue from previously conducted animal studies were used. All samples were obtained from studies conducted under Institutional Animal Care and Use Committee (IACUC)-approved protocols (1381MF (approved 14 June 2013), 1560MF (approved 20 September 2016)) in compliance with the Animal Welfare Act and other federal statutes and regulations relating to animals and experiments involving animals. Texas Biomedical Research Institute (TBRI) is accredited by the Association for Assessment and Accreditation of Laboratory Animal Care International and adheres to principles stated in the 8th Edition of the Guide for the Care and Use of Laboratory Animals, National Research Council [6]. Prior to any blood collections, animals were anesthetized using Telazol (Zoetis Inc., Parsippany-Troy Hills, NJ, USA), and euthanasia criteria were followed to minimize pain and distress; animals were euthanized with an intravenous overdose of sodium pentobarbital [7].

### 2.2. Cells and Virus

Biodefense and Emerging Infections Research Resources Repository (BEI Resources)-sourced Vero E6 cells (African green monkey kidney cell origin) were grown in Minimum Essential Media (MEM; Gibco, Grand Island, NY, USA) containing 2 mM l-glutamine (Gibco, Grand Island, NY, USA) and 1 mM sodium pyruvate (Gibco, Grand Island, NY, USA) (referred to as normal growth media) with 10% heat-inactivated fetal calf serum (FCS; Gibco, Grand Island, NY, USA) at 37 °C with 5% CO_2_ [7]. To demonstrate the limit of detection of the cell culture assay, generating EBOV-infected cells and inactivation of clarified cell culture media, a recombinant *Zaire ebolavirus* containing GFP (eGFP-ZEBOV, kindly provided by Dr. Heinz Feldmann) was used. To validate inactivation of virus in fluids from EBOV-exposed NHPs, serum collected from a cynomolgus macaque immediately prior to euthanasia during a previous animal study was used. To validate inactivation of virus in tissue from EBOV-exposed NHPs, liver collected from two cynomolgus macaques during necropsy during a previous animal study was used.

### 2.3. Generation of EBOV-Infected Cells for Use during Inactivation

Vero E6 cells were maintained in normal growth media with 10% FCS at 37 °C and 5% CO_2_. On the day before infection, cells were seeded in T150 flasks with 7 × 10^6^ cells per flask. On the morning of the infection, one flask was sacrificed to determine the number of cells (which ranged from 1 × 10^7^–1.4 × 10^7^). Inside the BSL4 laboratory, eGFP-ZEBOV virus was diluted in normal growth media with 2% FCS to achieve a multiplicity of infection (MOI) of 0.1 for the first validation experiment or 0.01 for the second validation experiment. Media were removed from flasks and replaced with 30 mL of the diluted virus. Cells were incubated at 37 °C and 5% CO_2_ for six days. The number of cells remaining on the day of harvest was estimated to be between 8 × 10^6^ and 8.9 × 10^6^ (based on confluency of cells in the flask and the number of cells estimated to be in a 100% confluent flask [8]). At time of harvest, cell culture media were removed from each flask and discarded. A cell scraper was used to remove the cells from the flask, and they were deposited into a 1.5-mL tube. The cells were pelleted by centrifugation at 3000× *g* for 5 min in a 5424R refrigerated centrifuge (Eppendorf, Hauppauge, NY, USA), and any remaining liquid was removed.

### 2.4. Generation of Transmission Electron Microscopy Grids for Inactivation

Electron microscopy grid preparation was carried out in the BSL4 laboratory. Viral supernatant (2 μL) was deposited directly onto carbon-formvar-coated 300-mesh grids (Polysciences, Warrington, PA, USA) and allowed to dry. The grids were fixed with 2% glutaraldehyde solution for 20 min (unless otherwise stated) and then rinsed by inversion on three sequential drops of ultrapure water. The fixative solution was 2% cacodylate-buffered glutaraldehyde solution prepared using a 0.4 M cacodylate buffer stock combined with 25% glutaraldehyde (Sigma-Aldrich, St. Louis, MO, USA) and ultrapure water.

### 2.5. Chemical Inactivation of Filovirus Infected Samples

#### 2.5.1. TRIzol LS Reagent

TRIzol LS reagent (Life Technologies, Carlsbad, CA, USA) was used to inactivate liquid samples (clarified cell culture media and serum harvested from a viremic NHP) containing EBOV. In a 1.5-mL tube, 250 µL of fluid were added to 750 µL TRIzol LS (ratio of 1:3, following the manufacturer’s instructions). The tube was shaken vigorously by hand for five seconds and incubated for 5 min at room temperature. As a positive control, 250 µL of the NHP serum was added to 750 µL of normal growth media containing 2% FCS, rather than TRIzol LS reagent. This positive control sample was used to inoculate cells to show CPE. It was also subjected to plaque assay to determine the maximum titer inactivated.

#### 2.5.2. TRIzol Reagent

TRIzol reagent (Life Technologies, Carlsbad, CA, USA) was used to inactivate tissue or cells containing EBOV. For tissue, a piece of frozen NHP liver tissue was thawed, weighed and cut down to achieve a weight of 100 mg. The 100 mg piece was placed into a 1.5-mL tube with a steel bead (Qiagen, Hilden, Germany) and 1 mL TRIzol reagent. Using a Qiagen TissueLyser, the tube was homogenized for 3 min at 30 Hz; the sample block holding the tube was rotated to ensure uniform homogenization; and the tube was homogenized again for 3 min at 30 Hz. After homogenization, the material in the tube resembled a slurry with no large chunks remaining. As a positive control, one piece of tissue was prepared as described, but resuspended in 1 mL of normal growth media with 2% FCS, rather than TRIzol reagent. This tube was then homogenized as described above. The positive control sample was used to inoculate cells to show CPE. It was also subjected to the plaque assay to determine the maximum titer inactivated.

For cells, a cell pellet was subjected to three cycles of freezing and thawing, and then, cells were resuspended in 1 mL TRIzol reagent. Another cell pellet was resuspended in 1 mL TRIzol reagent without any freezing or thawing. As a positive control, one cell pellet was subjected to three cycles of freezing and thawing, and then, cells were resuspended in 1 mL of normal growth media with 2% FCS. The positive control sample was used to inoculate cells to show CPE and green fluorescence. It was also subjected to the plaque assay. For the first validation, the back titer indicated that the cell pellet contained 5 × 10^7^ PFU, and for the second validation, the back titer indicated that the cell pellet contained 3.25 × 10^7^ PFU.

#### 2.5.3. RNA-Bee Reagent

RNA-Bee reagent (Tel-test, Friendswood, TX, USA) was used to inactivate bodily fluid samples containing EBOV. In a 1.5 mL tube, 100 µL of serum were added to 900 µL of RNA-Bee. The tube was shaken vigorously by hand for five seconds and incubated for 5 min at room temperature. As a positive control, 100 µL of serum were resuspended in 900 µL of normal growth media with 2% FCS, rather than RNA-Bee reagent. The positive control sample was also used to inoculate cells.

#### 2.5.4. Ten Percent Neutral Buffered Formalin

Ten percent neutral buffered formalin (Sigma-Aldrich, St. Louis, MO, USA) was used to inactivate tissue containing EBOV. At necropsy, liver tissue, no thicker than 6 mm and no larger than 25 mm, was taken and placed in a cassette. The cassette was placed into a container with 250 mL 10% neutral buffered formalin. The cassette was submerged for a maximum of 7 days (45 h during the first validation or 7 days for the second and third validations). Prior to validation of inactivation, the tissue was removed from the cassette and placed into a 1.5-mL tube with a steel bead and 1 mL normal growth media containing 2% FCS. The tube was homogenized once for 3 min at 30 Hz and then homogenized again for 3 min at 30 Hz. As a positive control, one piece of tissue was prepared as described above, but with no formalin incubation step. The positive control sample was used to inoculate cells to show CPE. It was also subjected to the plaque assay to determine the maximum titer inactivated.

#### 2.5.5. Osmium Tetroxide

Osmium tetroxide was used to inactivate transmission electron microscopy (TEM) grids carrying dried-down EBOV. A 1% osmium tetroxide solution was prepared from aqueous 2% osmium tetroxide (Sigma-Aldrich, St. Louis, MO, USA) diluted in ultrapure water. After preparation of TEM grids as described above, a piece of Kimwipe or paper towel was placed in the bottom of a 1.5 mL tube with a screw cap lid. A small volume, 50–100 µL of 1% osmium tetroxide solution, was added onto the paper (the paper became visibly damp, but not completely soaked). To suspend the grid above the liquid and in the vapors, a pipette tip was used. Scissors were used to cut off the widest part of a pipette tip (the tip was short enough to fit inside the 1.5-mL tube). The tip was carefully placed (with the point side down, widest part at the top of the tube) into the tube containing the osmium tetroxide-soaked paper. Using tweezers, the grid was then carefully placed inside the opening of the tip base. The tube was closed securely and remained closed for 1 h. Alongside inactivated samples, a positive control was included to visually confirm osmium tetroxide action. Following the procedure for inactivating the grids, osmium tetroxide-soaked paper was placed into a tube, and a trimmed pipette tip was placed inside. Rather than inserting a grid into the tip, a drop of viral supernatant was placed into the tip opening and the lid closed tightly. Over time, if the osmium tetroxide was creating vapors in the tube, the liquid viral supernatant would begin to change color, eventually becoming black. In addition to the prepared grids, positive and negative control samples were included: two 2-μL samples of the EBOV virus used during grid preparation were suspended on the ends of two inverted p200 micropipette tips. One tip was then incubated inside a 1.5-mL tube for one hour at room temperature in the absence of osmium tetroxide. The other tip was incubated inside a 1.5-mL tube for one hour at room temperature in the presence of 1% osmium tetroxide. Two grids were also prepared with the glutaraldehyde fixative step omitted. One positive control grid was prepared without exposure to glutaraldehyde or osmium tetroxide.

### 2.6. Determination of Viral Titers

Samples were titered on the day of each assay to verify the viral load used for validation. The assay was a neutral red and agarose plaque assay, as previously described [9]. Briefly, Vero E6 cells were seeded on 6-well plates and incubated overnight at 37 °C and 5% CO_2_. On the day of validation, virus was serially diluted in normal growth media with 2% FCS, and 400 μL of the appropriate dilution were used to inoculate cells. Cells were incubated for one hour at 37 °C and 5% CO_2_, at which time, the virus was removed and cells overlaid with a primary overlay consisting of 2× Eagle’s Minimum Essential Medium (EMEM) (Lonza, Walkersville, MD, USA) media mixed with agarose for a final agarose concentration of 1% and a final FCS concentration of 4%. After 7 days, a secondary overlay containing neutral red was added to each well; the secondary overlay consisted of 2× EMEM media mixed with agarose for a final agarose concentration of 1%, a final FCS concentration of 4% and a final neutral red concentration of 4%. Plates were returned to the incubator for approximately 24 h, at which time plaques presented as clearings that could be counted with the naked eye. Plates were scanned on a flatbed scanner to capture plaques for counting. Wells with an intact monolayer and plaque numbers ranging from 10–150 were used to calculate the titer, and titers from replicate plates were averaged. The titer was calculated as: number of plaques counted in a well × 2.5 (to account for plating 400 μL per well) × dilution factor of well = PFU/mL.

### 2.7. Testing of Experimental Samples after Inactivation with TRIzol LS Reagent, TRIzol Reagent, RNA-Bee Reagent and Formalin

Following treatment with the inactivation reagent or mock treatment for positive controls, inoculation of Vero E6 cells was performed to validate that the inactivation procedure was successful. Vero E6 cells were inoculated in 500-cm^2^ dishes with the entire volume of the aforementioned test sample and 49 mL of normal growth media containing 2% FCS. A negative control (media + inactivation reagent, no virus) was included to permit comparison showing the appearance of cells with no virus present. Virus inoculum was added to cells and allowed to incubate for 1 h at 37 °C. After 1 h, the inoculum was removed and fresh normal growth media (containing 2% FCS) added to the cells. Cells were then incubated for 7 days at 37 °C. These cells were observed and imaged daily for a week. Cells inoculated with samples containing eGFP-ZEBOV (samples of infected cells and cell culture media) were observed for CPE and green fluorescence, while cells infected with all other sample types were observed for CPE only. After one week, all of the cell culture media were removed from each plate and used to inoculate fresh Vero E6 cells in a T225 flask; the entire volume of media from one plate was deposited into one flask. These cells were observed and imaged daily. This entire process was repeated independently on two (RNA-Bee and TRIzol reagent with infected cells) or three (TRIzol LS reagent, formalin and TRIzol reagent with tissue) separate occasions.

### 2.8. Testing of Experimental Samples after Inactivation with 1% Osmium Tetroxide

Following treatment with 1% osmium tetroxide, grids and control tips were incubated with 1.5 mL normal growth media containing 2% FCS at 37 °C with 5% CO_2_ for one hour. Three samples of media were also incubated with 2 μL of the EBOV as a positive control. After incubation, each 1.5-mL sample was used to overlay a corresponding well of Vero E6 cells on a six-well cell culture plate (previously seeded with 6–9 × 10^5^ cells per well). A well of cells was also overlaid with mock infected media. Cells were viewed after infection to look for CPE and the presence of green fluorescence that would indicate viral infection. Images of cells were taken every other day for eight days, using identical light settings and 4× magnification on the fluorescent microscope. After eight days, the cell culture media from each well were collected and used to inoculate another set of Vero E6 cell culture plates. These cells were then also monitored post inoculation and observed for CPE and the presence of green fluorescence. This process was repeated independently on two occasions.

### 2.9. Determination of Limit of Detection

Recombinant EBOV that expresses GFP (eGFP-ZEBOV) was serially diluted in normal growth media with 2% FCS to produce material containing 10 PFU, 1 PFU and 0.1 PFU in 50 mL. The stock virus was back titered during the test to verify the inocula titers. Each dilution was added to Vero E6 cells in 500-cm^2^ dishes and allowed to incubate for 1 h at 37 °C. After 1 h, the inoculum was removed and fresh normal growth media with 2% FCS added to the cells. Cells were then incubated for 7 days at 37 °C. Photos were taken on Day 7 to demonstrate that a very low quantity of infectious virus would cause green fluorescence and observable cytopathic effect (CPE) such as cell rounding, cell detachment and membrane blebbing. After imaging, the cell culture media were blind-passaged on naive Vero E6 cells in T225 flasks, and after 7 days, images were again taken. This process was repeated three times. Based on the back titers, the inoculum for each experiment were as follows: first validation: back titer 1.75 × 10^7^ PFU/mL, inocula: 18 PFU, 2 PFU, 0.2 PFU; second validation: back titer 5 × 10^7^ PFU/mL, inocula: 50 PFU, 5 PFU, 0.5 PFU; third validation: back titer 3.25 × 10^7^ PFU/mL, inocula 33 PFU, 3 PFU, 0.3 PFU.

### 2.10. Readout for the Presence of Infectious Virus

Cells were observed throughout incubation during the initial infection and subsequent blind passage to monitor for the appearance of CPE and, when applicable, green fluorescence. Cells inoculated with samples containing eGFP-ZEBOV (samples of infected cells and cell culture media) were observed for CPE and green fluorescence, while cells infected with all other sample types were observed for CPE only. Limit of detection samples containing a very low quantity of infectious virus (described in Section 2.9) were processed each time a validation occurred to allow for monitoring of the frequency and appearance of CPE resulting from fewer than 5 PFU. Based on these samples, virally-induced CPE was defined as patches of cell rounding, cell detachment and membrane blebbing. Imaging was performed using a 4× objective on an EVOS digital fluorescent microscope (Advanced Microscopy Group, Mill Creek, WA, USA). Transmitted light images were viewed with 40 percent illumination, and fluorescent images were viewed on the green fluorescent protein (GFP) channel with 60 percent illumination.

## 3. Results

### 3.1. Establishing an Assay for Testing Infectivity

Many of the chemicals used for routine inactivation are cytotoxic. This makes it difficult to establish an in vitro assay to demonstrate the lack of infectious virus in potentially inactivated samples. Further, demonstrating complete inactivation of an entire sample using only a portion of each inactivated sample is undesirable. Thus, it was necessary to develop an in vitro method that would allow for the demonstration of a lack of infectious virus in the entirety of a potentially inactivated sample without causing excessive cytotoxicity. Mock infected samples of cell culture media were mixed at an appropriate ratio with each inactivation reagent in a total volume of 1 mL. These samples were added to Vero E6 cells and incubated for one hour at 37 °C. After one-hour of incubation, the mixture was removed, and fresh cell culture media were added. Cells were monitored over the next week for signs of cytotoxicity as evidenced by CPE. It was determined that negligible CPE was present in cells incubated with 1 mL inactivated mock-infected samples mixed with 49 mL cell culture media. Typically, our procedures call for a low ratio of surface area to inoculum volume during the initial one-hour incubation period, prior to inoculum being removed and replaced with fresh growth media. To achieve a correspondingly low inoculum volume to surface area ratio when using 50 mL total inoculum, large cell culture dishes were required. As such, 500-cm^2^ culture dishes were chosen for use during infectivity testing of potentially inactivated samples. Crucially, increasing this volume and dish size did not reduce the sensitivity of the infectivity-based read-out assays.

In addition, an experiment was performed where cells were inoculated with media containing TRIzol LS (49 mL media plus 750 μL TRIzol LS plus 250 μL PBS) for one hour. The inoculum was removed and replaced with fresh growth media, and cells were infected with a low amount of eGFP-ZEBOV. These cells developed extensive CPE and green fluorescence, indicating that exposure to the diluted TRIzol LS reagent did not inhibit the ability of the cells to support viral replication.

### 3.2. Determination of Limit of Detection

We used CPE and, when possible, green fluorescence in Vero E6 cells as a readout for the presence of infectious virus during infectivity testing of potentially inactivated samples. We determined the limit of detection for this readout using recombinant EBOV expressing GFP (eGFP-ZEBOV). Virus was diluted such that approximately 10, 1 and 0.1 PFU were represented to show CPE and green fluorescence following infection by small quantities of infectious virus. After seven days, cells exposed to approximately 10 or 1 PFU of virus exhibited clear evidence of CPE and green fluorescence, while cells exposed to approximately 0.1 PFU exhibited no CPE or green fluorescence. When this material was passaged, thus amplifying the virus further, the degree of CPE and green fluorescence intensified. Thus, in the Vero E6 cell culture, less than five PFU of EBOV was sufficient to cause visible CPE. As such, this is a viable readout for determining the presence of infectious virus.

### 3.3. Chemical Inactivation of Ebola Virus

Ebola virus was prepared in the BSL4 to inactivate all infectious virus in various different matrices using various chemical methods (see Table 1 for a summary of matrices and reagents). Briefly, prior to being considered inactivated and tested for infectious virus:

- Clarified cell culture media were mixed with TRIzol LS reagent, following the manufacturer’s instructions.
- Serum was mixed with TRIzol LS reagent or with RNA-Bee reagent.
- Tissue was submerged in 10% neutral buffered formalin, or mixed with TRIzol reagent and a steel bead and homogenized.
- Cells were pelleted and resuspended in TRIzol reagent.

### 3.4. Testing of Experimental Samples after Inactivation with TRIzol LS Reagent, TRIzol Reagent, RNA-Bee Reagent and Formalin

Following treatment with the inactivation reagents, inoculation of Vero E6 cells was performed to validate successful inactivation. Vero E6 cells were inoculated with the aforementioned test samples and monitored for CPE (and green fluorescence when applicable). A negative control was included for each inactivation reagent (no virus, but media plus appropriate reagent) to represent cell appearance with no virus present. For each type of sample inactivated, a corresponding mock inactivated sample was also plated on Vero E6 cells as a positive control. To determine the limit of detection, eGFP-ZEBOV of the known titer was included to represent what expected CPE and green fluorescence would look like from approximately 10, 1 and 0.1 PFU, as described above. Cells were observed for CPE (and also green fluorescence in samples containing eGFP-ZEBOV) and imaged for one week. After one week, cell culture media were blind passaged onto fresh Vero E6 cells. A positive control containing a low quantity of virus was again included. These cells were observed and imaged, as well. If there were a single infectious virus particle in the “inactivated” material, it should infect cells and be amplified. Virus in passage 1 should be further amplified during the second blind passage; this is supported by the positive control using approximately one PFU. All positive control wells developed extensive CPE (cell rounding, cell detachment, membrane blebbing). Positive control samples containing eGFP-ZEBOV (samples of infected cells or cell culture media) also showed extensive green fluorescence. None of the inactivated samples resulted in the appearance of appreciable CPE, and inactivated samples containing eGFP-ZEBOV (samples of infected cells or cell culture media) also exhibited no green fluorescence. All were indistinguishable from the corresponding negative controls containing no virus, but media plus the appropriate reagent.

### 3.5. Testing of Experimental Samples after Inactivation with 1% Osmium Tetroxide

After optimization of electron microscopy (EM) grid preparation methods, the osmium tetroxide negative staining step needed to be validated to ensure it served as an adequate inactivation step. The aim was to verify that the EM grids and any agents also present in a tube containing a suspended grid could be properly inactivated using 1% osmium tetroxide vapors. Grids were prepared as described in the Methods section (and previously [10]) and were exposed to: both 2% glutaraldehyde and 1% osmium tetroxide, only 1% osmium tetroxide, or neither glutaraldehyde, nor osmium tetroxide. In addition, p200 micropipette tips containing 2 μL (7 × 10^4^ PFU) of eGFP-ZEBOV virus were incubated with or without 1% osmium tetroxide. It was hypothesized that during grid preparation, suspending viral supernatant on an EM grid could compromise the viral envelope and thus decrease infectivity. It was important to demonstrate that the osmium tetroxide vapors alone were effective at eliminating infectivity in viral samples not suspended on EM grids. After preparation, the grids and tips were incubated with cell culture media that was then used to overlay Vero E6 cells. Cells were viewed after infection to look for cytopathic effects and the presence of green fluorescence that would indicate viral infection. Images of cells were taken every other day for eight days, using identical light and magnification settings on a fluorescent microscope. Following incubation of cells with media exposed to EM grids treated with 2% glutaraldehyde and 1% osmium tetroxide, or 1% osmium tetroxide, there was an absence of green fluorescent cells and no CPE. Incubation with media exposed to tips that contained EBOV virus and were treated with 1% osmium tetroxide also resulted in an absence of green fluorescent cells and no CPE. Cells inoculated with these media samples all appeared similar to the mock-infected cells. Conversely, grids and tips that were exposed to EBOV, but not to 1% osmium tetroxide resulted in green fluorescent cells and CPE. Cells inoculated with these media samples all appeared similar to the positive control infected cells. The absence of green (infected) cells following infection with media exposed to EM grids treated with 1% osmium tetroxide alone, 2% glutaraldehyde and 1% osmium tetroxide, or untreated suggested that contact with the grid alone was sufficient to inactivate EBOV. The absence of green cells or CPE when virus was exposed to osmium tetroxide in a tube, compared to the presence of green cells and CPE when identically-treated virus was not exposed to osmium tetroxide, is consistent with effective inactivation of the tube contents by osmium tetroxide.

## 4. Discussion

Material infected with FSAP-regulated viruses can only be moved to lower containment levels after being rendered non-infectious, which requires demonstration that the agent no longer has any ability to grow or replicate. This can be achieved by using well-established inactivation procedures, but the procedures must be validated in-house to confirm a lack of infectivity [4]. This study aimed to describe a method for validating chemical inactivation and to apply the method to demonstrate the effectiveness of various inactivation methods.

Samples containing EBOV were prepared in the BSL4 laboratory to inactivate all infectious virus in different matrices using various chemical methods. The presence of infectious virus was determined by assessing CPE, which we determined to be a viable readout for detecting even a very low quantity of infectious virus. We found that, when used following standard procedures as documented in the manufacturer’s instructions, TRIzol LS reagent (Life Technologies) and RNA-Bee (Tel-Test B Labs) inactivated EBOV in serum, TRIzol LS reagent (Life Technologies) inactivated EBOV in clarified cell culture media, TRIzol reagent (Life Technologies) inactivated EBOV in 100 mg of tissue, TRIzol reagent (Life Technologies) inactivated EBOV infected Vero E6 cells, 10% neutral buffered formalin (Sigma-Aldrich) inactivated EBOV in 1 g of tissue and 1% osmium tetroxide (Sigma-Aldrich) inactivated EBOV dried down on TEM grids.

At times, it may be necessary to inactivate larger volumes of biological fluids or larger pieces of tissue than described herein. While users must work with regulators and their institutional committees to ensure compliance with inactivation requirements, the inactivation methods described can be scaled up easily. For example, TRIzol LS and TRIzol reagents can both be used with larger quantities of sample as long as the proper ratio of sample to reagent is maintained (e.g., 1 mL of fluid can be treated with 3 mL of TRIzol LS reagent) and an appropriately sized vessel is used to minimize dead space. Validation might be performed by splitting larger samples into smaller volumes for infectivity testing or by using a high titer sample such that the quantity of infectious virus inactivated in 250 µL is greater than the quantity that would be present in the 1 mL requiring inactivation. Formalin inactivation of larger pieces of tissue should also be effective. Validation of proper inactivation of larger tissue pieces could be performed by dividing a larger section into smaller sections, after inactivation, to test for infectious virus.

It should be noted that this methodology applies to EBOV; the requirements for inactivation and validation of inactivation may differ for other agents or matrices. It is the responsibility of each institution and user to understand and follow the FSAP regulations for the specific agent used.

Previous studies have demonstrated various inactivation methods for EBOV [11,12,13,14,15]. However, this study expands upon previous reports in a number of ways. The method described herein allows for testing inactivation with reagents that can cause cytotoxicity and eliminates the need for dialysis (or other similar methods) to remove these inactivating reagents. In addition, this study included validation of a wide range of reagents that were not previously documented as being well validated, including TRIzol LS reagent, RNA-Bee reagent and one hour exposure to osmium tetroxide vapors.

The methods described herein can be easily employed by other groups to validate the inactivation of filoviruses in various matrices and by various chemical methods, in order to facilitate work at lower containment levels.

## Figures and Tables

**Table 1:** Summary of inactivation methods. EBOV, *Zaire ebolavirus*.

Inactivation Reagent	Type of Sample Inactivated	Volume of Inactivation Reagent	Quantity of Sample Inactivated	Inactivation Incubation Time	Maximum PFU ^1^ Inactivated
TRIzol LS reagent	Clarified cell culture media (containing eGFP-ZEBOV ^2^)	750 µL	250 µL	5 min, RT ^3^	1.25 × 10^7^ PFU
	Bodily fluid (e.g., serum)	750 µL	250 µL	5 min, RT	2.45 × 10^8^ PFU
TRIzol reagent	Infected cells (containing eGFP-ZEBOV)	1 mL	≤8 × 10^6^ cells	5 min, RT	7 × 10^7^ PFU
	Tissue	1 mL	100 mg	Homogenized, 3 min at 30 Hz, two times, RT	7.7 × 10^7^ PFU
RNA-Bee reagent	Bodily fluid (e.g., serum)	900 µL	100 µL	5 min, RT	2.4 × 10^7^ PFU
10% neutral buffered formalin	Tissue	250 mL	≤1 g	≤1 week	1.2 × 10^9^ PFU
1% osmium tetroxide	TEM ^4^ grids (containing eGFP-ZEBOV)	50–100 µL	1 grid	60 min	7 × 10^4^ PFU

^1^ PFU, plaque forming unit. ^2^ eGFP-ZEBOV, recombinant *Zaire ebolavirus* containing GFP, ^3^ RT, room temperature; ^4^ TEM, transmission electron microscopy.
